# Audiovisual temporal processing in adult patients with first-episode schizophrenia and high-functioning autism

**DOI:** 10.1038/s41537-022-00284-2

**Published:** 2022-09-22

**Authors:** Han-yu Zhou, Iris Y. S. Lai, Karen S. Y. Hung, Mandy K. M. Chan, Zoe T. Y. Ho, Jenny P. H. Lam, Simon S. Y. Lui, Raymond C. K. Chan

**Affiliations:** 1 Shanghai Key Laboratory of Mental Health and Psychological Crisis Intervention, School of Psychology and Cognitive Science, East China Normal University, Shanghai, China; 2 Castle Peak Hospital, Hong Kong Special Administrative Region, Hong Kong, China; 3 Department of Psychiatry, School of Clinical Medicine, The University of Hong Kong, Hong Kong Special Administrative Region, Hong Kong, China; 4 Neuropsychology and Applied Cognitive Neuroscience Laboratory, CAS Key Laboratory of Mental Health, Institute of Psychology, Chinese Academy of Sciences, Beijing, China; 5 Department of Psychology, University of Chinese Academy of Sciences, Beijing, China

**Keywords:** Developmental biology, Biomarkers

## Abstract

Schizophrenia and autism spectrum disorder (ASD) are both neurodevelopmental disorders with altered sensory processing. Widened temporal binding window (TBW) signifies reduced sensitivity to detect stimulus asynchrony, and may be a shared feature in schizophrenia and ASD. Few studies directly compared audiovisual temporal processing ability in the two disorders. We recruited 43 adult patients with first-episode schizophrenia (FES), 35 average intelligent and verbally-fluent adult patients with high-functioning ASD and 48 controls. We employed two unisensory Temporal Order Judgement (TOJ) tasks within visual or auditory modalities, and two audiovisual Simultaneity Judgement (SJ) tasks with flash-beeps and videos of syllable utterance as stimuli. Participants with FES exhibited widened TBW affecting both speech and non-speech processing, which were not attributable to altered unisensory sensory acuity because they had normal visual and auditory TOJ thresholds. However, adults with ASD exhibited intact unisensory and audiovisual temporal processing. Lower non-verbal IQ was correlated with larger TBW width across the three groups. Taking our findings with earlier evidence in chronic samples, widened TBW is associated with schizophrenia regardless illness stage. The altered audiovisual temporal processing in ASD may ameliorate after reaching adulthood.

## Introduction

Atypical sensory processing is associated with schizophrenia^1^ and autism spectrum disorder (ASD)^2^, and is believed to be one possible neurobiological mechanism for schizophrenic symptoms such as hallucinations and disorganization^3^ and ASD features such as social communicative impairments^4^. Multisensory integration refers to the processing and binding of perceptual information of different sensory modalities together to generate a coherent percept, and is one of the important aspects in sensory processing^5^. During multisensory integration, spatial and temporal information are essential for the brain to determine whether the two sensory stimuli should be bound together as a unified percept^6^. Due to the physical properties of sensory stimuli (i.e., the speed of light versus the speed of sound) and the physiological properties of neuron transmissions, even multimodal stimuli coming from the same external source are asynchronous signals in the physical world. For instance, when both auditory and visual stimuli emerge at the same time from the same spot of source, the auditory signal always lags behind the visual signal. The human neural system has built-in mechanisms to adapt to this natural delay in signals for appropriate multisensory integration, a phenomenon called “temporal binding window” (TBW)^7^.

Experimental research on multisensory integration and TBW in patients with ASD and patients with schizophrenia has attracted growing interest (see review ref. ^8^). Most previous studies in this area recruited children and adolescents with ASD^9–12^ or adults with chronic schizophrenia (e.g. refs. ^13–15^). Few studies on multisensory integration recruited adults with ASD^16,17^. The results generally supported that both schizophrenia and ASD are associated with reduced sensitivity of detecting audiovisual asynchrony, manifested as an abnormally widened TBW^18^. Difficulties in audiovisual temporal processing have been found to affect both speech and non-speech processing in patients with schizophrenia, whereas it remains unclear whether patients with ASD would exhibit altered integration of non-speech stimuli^8^.

It is noteworthy that most previous studies on temporal processing utilized either ASD sample or schizophrenia sample alone (see ref. ^8^ for a review). Little is known about the differences and similarities in TBW between these two clinical entities. Given the shared social difficulties in ASD and schizophrenia^19^, and the important role of audiovisual temporal integration in scaffolding social and communicative functions^20–22^, it is worthwhile to directly compare the two clinical conditions and to examine whether widened TBW would contribute to high levels of social and cognitive difficulties. One study administered the speech-related simultaneous judgement (SJ) task to adolescents with ASD and adults with schizophrenia, which measured multisensory temporal integration^23^. Another recent study investigated both the unisensory and multisensory temporal processing in children with ASD and adolescents with early onset schizophrenia, and the results revealed generalized difficulties in temporal processing in schizophrenia, but ASD was associated with temporal processing differences affecting only speech stimuli^24^. In both studies^23,24^, samples of the two disorders were recruited separately, and comparison between the two disorders in the TBW was only conducted in a post-hoc manner, and the results were confounded by the unmatched demographics of the two clinical groups. Moreover, considering the drastic developmental changes of TBW during childhood and adolescence^8^, whether the differences of temporal sensory processing in patients with ASD and patients with schizophrenia would change as they reach adulthood is of great research interest.

By contrast, this study directly compared adult patients with high-functioning ASD with adult patients with first-episode schizophrenia (FES) in terms of audiovisual temporal processing. We attempted to address the other limitations of previous studies, such as failure to include adult samples with ASD^25^, use of samples with chronic schizophrenia^14,15^, and failure to investigate both unisensory and multisensory integrations^23^. Therefore, this study utilized adult samples of ASD and FES, and demographically-matched controls. We also employed the unisensory Temporal Order Judgement (TOJ) tasks which measured unisensory temporal acuity in both auditory and visual processing; these variables would need to be accounted for while investigating multisensory temporal integration. Moreover, audiovisual SJ tasks were used to estimate TBW for both non-speech and speech stimuli. The objectives of this study were to comprehensively examine audiovisual temporal processing in adult patients with schizophrenia and ASD, and to make direct comparison of the two clinical disorders. Based on previous findings^24^, we hypothesized that both adult patients with ASD and adult patients with FES would exhibit a widened TBW for audiovisual speech stimuli relative to controls, but only adult patients with FES would have difficulty in temporally integrating simple non-speech auditory and visual inputs.

## Results

Table 1 shows the characteristics of our sample. The two clinical groups and controls were matched in age and gender (*p* > 0.05). However, estimated non-verbal IQ in the FES group was significantly lower than that in the ASD group (*p* = 0.022) and controls (*p* < 0.001). The controls had longer years of education than both clinical groups (*p*s < 0.001), whilst the FES group had longer years of education than the ASD group (*p* < 0.05).

**Table 1:** Summary of demographic and clinical information of participants.

	FES (*n* = 43)		ASD (*n* = 35)		Controls (*n* = 48)		*F*_(2,123)_/*χ*^2^/*t*	*p*-values
	Mean	SD	Mean	SD	Mean	SD		
Age (years)	25.93	5.216	23.83	3.944	25.02	3.784	2.236	0.111
Gender ratio (male vs female)	27 vs 16 (1.69)		27 vs 8 (3.38)		30 vs 18 (1.67)		2.394	0.302
Education (years)	13.91	3.01	12.49	1.60	17.02	1.78	44.32	<0.001
Estimated IQ (TONI-4)	96.93	11.774	104.26	13.842	108.13	10.139	10.365	<0.001
PANSS positive symptoms	8.21	2.678						
PANSS negative symptoms	11.49	4.857						
PANSS general symptoms	18.30	3.635						
ESRS	0.70	1.64	0.00	0.00			4.105	<0.001
DDD (mg/day, olanzapine equivalence), (FES: *n* = 43; ASD: *n* = 11)	14.24	8.40	3.63	2.85			1.335	0.188

*FES* first-episode schizophrenia, *ASD* autism spectrum disorder, *PANSS* The Positive and Negative Syndrome Scale, *ESRS* Extrapyramidal Symptom Rating Scale, *DDD* Defined Daily Dose (mg/day).

### Unisensory temporal acuity

**Fig. 1:**
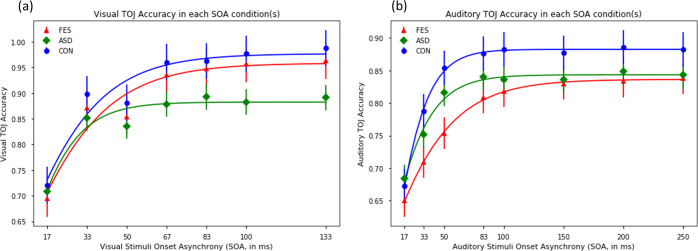
The results of Unisensory Temporal Order Judgement (TOJ) tasks in the three groups. The results of visual (**a**) and auditory (**b**) TOJ tasks. FES first episode schizophrenia, ASD autism spectrum disorder, CON controls. The bars indicate standard errors at each SOA.

Figure 1 illustrates the performance of TOJ tasks (Fig. 1a for visual TOJ; Fig. 1b for auditory TOJ) at different Stimulus Onset Asynchrony (SOA) conditions in the three groups. Regarding the visual TOJ task, the mixed model ANOVA showed that the Group main effect (*F*_[2,123]_ = 2.772, *p* = 0.066, partial-*η*^2^ = 0.043) and the Group-by-SOA condition interaction (*F*_[12,738]_ = 2.112, *p* = 0.056, partial-*η*^2^ = 0.033) failed to reach statistical significance. We conducted post-hoc analyses to clarify the Group-by-SOA condition interaction effect which had a trend of statistical significance. The post-hoc results showed that ASD participants, compared with the other two groups, had lower accuracy to judge the temporal order of visual stimuli at large SOA conditions (Supplementary Table 1). A closer examination of the data on the within-group variations showed that 4 participants in the ASD group had very low accuracy (below random guess, <50%) to judge temporal order even at the largest SOA. As expected, the SOA condition main effect was significant (*F*_[6,738]_ = 146.425, *p* < 0.001, partial-*η*^2^ = 0.543), suggesting participants’ higher accuracy in judging temporal orders when SOAs increased.

For the auditory TOJ task, 1 FES participant failed to meet the minimum requirements (i.e., >75% accuracy) in the practice trials and thus was excluded. The Group main effect (*F*_[2,122]_ = 0.909, *p* = 0.406, partial-*η*^2^ = 0.015) and the Group-by-SOA condition interaction (*F*_[14,854]_ = 0.914, *p* = .469, partial-*η*^2^ = 0.015) were not significant. Moreover, the TOJ accuracy in each of the SOA conditions in the auditory TOJ task did not differ between the three groups of participants (Supplementary Table 2). However, the SOA condition main effect reached statistical significance (*F*_[7,854]_ = 55.795, *p* < 0.001, partial-*η*^2^ = 0.31).

Regarding the TOJ thresholds, the TOJ accuracy for each SOA was fitted into a sigmoid curve on a participant-by-participant basis, and the SOA at which a participant could attain 75% of accuracy was estimated as a “proxy index” for his/her “unisensory temporal acuity”^26^. After excluding participants (visual TOJ: *n* = 2 for FES participants, *n* = 6 for ASD participants, *n* = 3 for controls; auditory TOJ: *n* = 6 for FES participants, *n* = 6 for ASD participants, *n* = 6 for controls) whose data fitted poorly because of their extremely low accuracy (<−2 SD below the mean) at the largest SOA condition, the ANOVA results did not find any significant group difference in both the visual (*F*_[2,112]_ = 1.39, *p* = 0.253) and auditory (*F*_[2,104]_ = 0.719, *p* = 0.49) task (see Table 2). After excluding participants who had poor data fitting or had failed the minimum requirements in the practice trials, the three groups did not differ in age and gender ratio (*p*s > 0.05), except for the age differences when comparing auditory TOJ threshold (*F*_[2, 104]_ = 3.23, *p* = 0.044; post-hoc: FES > ASD) (see Supplementary Materials). Therefore, in addition, we carried out ANCOVA analysis with age as a covariate to compare the auditory TOJ threshold between the three groups. The covariate analysis showed the same results, suggesting comparable auditory TOJ thresholds across the three groups (*F*_[2,103]_ = 0.858, *p* = 0.430). Taken together, participants with FES, participants with ASD and controls had comparable levels of unisensory temporal acuity for processing visual and auditory stimuli.

**Table 2:** Group difference in TOJ thresholds and TBW width.

POST-HOC (SIDAK) COMPARISON											
	FES		ASD		Controls				FES vs. CON	ASD vs. CON	FES vs. ASD
	Mean	SD	Mean	SD	Mean	SD	*F*	*p*	*p*	*p*	*p*
Visual TOJ threshold	8.74	46.19	22.56	31.96	18.82	29.30	1.390	0.253	0.501	0.964	0.329
	(*n* = 41)		(*n* = 29)		(*n* = 45)						
Auditory TOJ threshold	88.0	272.51	97.79	283.73	34.05	197.73	0.719	0.490	0.708	0.645	0.998
	(*n* = 36)		(*n* = 29)		(*n* = 42)						
Flash-beep TBW	361.50	128.98	305.71	199.58	276.38	98.96	3.651	0.029*	0.024*	0.754	0.311
	(*n* = 34)		(*n* = 30)		(*n* = 48)						
Flash-beep PSS	37.95	74.14	62.42	99.09	31.09	77.48	1.367	0.259	0.713	0.107	0.241
	(*n* = 34)		(*n* = 30)		(*n* = 48)						
Syllable TBW	391.70	179.69	319.96	146.71	305.40	114.37	3.493	0.034*	0.035*	0.965	0.164
	(*n* = 31)		(*n* = 29)		(*n* = 46)						
Syllable PSS	−101.17	109.65	−78.49.96	112.39	−75.13	78.58	0.707	0.496	0.256	0.885	0.373
	(*n* = 31)		(*n* = 29)		(*n* = 46)						

*FES* first-episode schizophrenia, *ASD* autism spectrum disorder, *CON* healthy controls, *TOJ* temporal order judgement, *PPS* Point of Subjective Simultaneity, *TBW* Temporal Binding Window.

*Indicates *p* < 0.05; all measurements are in milliseconds (ms). The number of participants in each group after excluding those with poor data fitting was indicated in the blanket.

### Audiovisual temporal binding windows

**Fig. 2:**
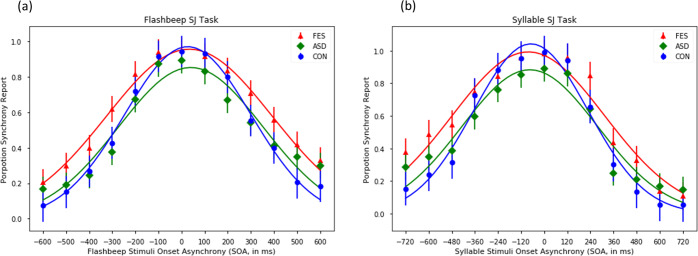
The results of Audiovisual Simultaneity Judgement (SJ) tasks in the three groups. The results of flash-beep (**a**) and syllable (**b**) SJ tasks. *FES* first episode schizophrenia, *ASD* autism spectrum disorder, *CON* controls. The bars indicate standard errors at each SOA.

Figure 2 shows the performances of flash-beep (Fig. 2a) and syllable (Fig. 2b) SJ tasks at different SOA conditions for the clinical groups and controls. In the flash-beep SJ task, 8 FES participants failed to meet the minimum requirement of 75% accuracy in the practice trials, and therefore were excluded from undergoing the SJ tasks. The mixed model ANOVA found that the Group main effect (*F*_[2,115]_ = 7.228, *p* = 0.001, partial-*η*^2^ = 0.112), the SOA condition main effect (*F*_[12,1380]_ = 260.721, *p* < 0.001, partial-*η*^2^ = 0.694), and the Group-by-SOA condition interaction (*F*_[24,1380]_ = 3.004, *p* = 0.002, partial-*η*^2^ = 0.050) all reached statistical significance. Post-hoc analysis indicated that FES participants, compared with controls, were more likely to perceive synchrony at large SOAs (see Supplementary Table 3).

In the syllable SJ task, 11 FES participants, 1 ASD participants and 2 controls failed to meet the minimum requirements in the practice trials and therefore were excluded. The mixed model ANOVA found that the Group main effect (*F*_[2,109]_ = 4.610, *p* = 0.012, partial-*η*^2^ = 0.078), the SOA Condition main effect (*F*_[12,1308]_ = 238.436, *p* < 0.001, partial-*η*^2^ = 0.686), and the Group-by-SOA Condition interaction (*F*_[24,1308]_ = 3.546, *p* < 0.001, partial-*η*^2^ = 0.061) all reached statistical significance. Specifically, FES participants had a higher tendency to report speech synchrony even at large SOAs (e.g., −720 ms, −600 ms) as indicated by post-hoc analysis results (Supplementary Table 4).

To estimate the width of the audiovisual TBW, we fitted the percentage of simultaneity responses for each SOA in the SJ tasks to the Gaussian distribution on a participant-by-participant basis^24^. The standard deviation was extracted to indicate the width of TBW within which a participant would be highly likely to perceive two stimuli as synchronous. The mean of the Gaussian function indicated the SOA at which a participant would have the highest likelihood to perceive synchrony (i.e., the point of subjective simultaneity, PSS). Participants whose data fitted poorly to the Gaussian function (*R*^2^ < 0.3) were excluded from further analysis. Specifically, 1 FES participant and 5 ASD participants were excluded from subsequent estimations of the TBW for non-speech stimuli, and the same number of FEP and ASD participants were excluded from that of the TBW for speech stimuli. The remaining participants did not differ in age and gender ratio (*p*s > 0.05) (see Supplementary Materials). The ANOVA results showed that the three groups differed significantly in the TBWs for both non-speech (flash-beep) stimuli (*F*_[2,109]_ = 3.65, *p* = 0.029) and speech stimuli (*F*_[2,103]_ = 3.49, *p* = 0.034). Specifically, FES participants had a wider TBW relative to controls regardless of stimulus types (non-speech: *p* = 0.024, Cohen’s *d* = 0.74; speech: *p* = 0.035, Cohen’s *d* = 0.57), but ASD participants and controls showed comparable non-speech (*p* = 0.754) and speech (*p* = 0.965) TBW width. The TBW did not differ significantly between the two clinical groups (non-speech: *p* = 0.311; speech: *p* = 0.164). The PSS for both non-speech and speech stimuli was comparable across the three groups (see Table 2). Together, these results supported the presence of a widened audiovisual TBW regardless of stimulus type in FES participants rather than ASD participants.

### Correlations with non-verbal IQ and clinical characteristics

The results of Spearman’s correlations are shown in Supplementary Table 5. Across the three groups, non-verbal IQ was significantly correlated with TBW for non-speech stimuli (*r*_(112)_ = −0.409, *p* < 0.001), and TBW for speech stimuli (*r*_(106)_ = −0.329, *p* = 0.001). We conducted correlational analyses within each of the three groups, and the results showed that ASD participants with lower non-verbal IQ exhibited wider TBWs for both non-speech (flash-beep) stimuli (*r*_(30)_ = −0.416, *p* = 0.022) and speech stimuli (*r*_(29)_ = −0.380, *p* = 0.042). No significant correlation was found between temporal processing acuity and medications, levels of extrapyramidal symptoms, and clinical symptoms as measured by the PANSS in FEP participants (*p*s > 0.05).

## Discussion

This study is one of the few comprehensive investigations on the ability of unisensory and audiovisual temporal processing in adult patients with schizophrenia and adult patients with high-functioning ASD. Contrary to the majority of previous studies^9–15,24^, we utilized schizophrenia and ASD samples with comparable demographic features, and this method allowed direct comparisons of the two clinical groups to clarify the similarities and differences of unisensory and multisensory processing in patients with schizophrenia and patients with ASD. The key findings of this study are summarized as follows. First, FES patients exhibited widened TBW affecting both speech and non-speech processing, relative to controls. Second, the imprecise multisensory integration (i.e., widened TBW) in FES patients could not be attributable to unisensory differences, because they exhibited intact unisensory temporal processing and their TOJ thresholds were similar to healthy people. Third, participants (regardless of group status) having low estimated non-verbal IQ were more likely to have widened TBWs for processing speech and non-speech stimuli. Contrary to the findings in FES, adults with ASD exhibited comparable unisensory thresholds and audiovisual TBWs to healthy people, indicating their largely preserved ability of sensory temporal processing.

### Unisensory temporal acuity

FES patients showed intact ability to code the temporal order of sensory events in visual and auditory modalities, which is divergent from previous meta-analytic findings^18^, as well as a recent study concerning adolescents with early onset schizophrenia^24^. It is noteworthy that the studies included in Zhou et al.’s^18^ meta-analysis mainly recruited samples with chronic schizophrenia with long durations of illness, but this study recruited a young adult sample with FES. Age and cognitive functions have been found to influence an individual’s unisensory temporal acuity^27^, and may explain our divergent findings. Early-onset schizophrenia (for instance, the sample recruited in Zhou et al.’s^24^ study) is a relatively rare type of schizophrenia affecting less than 4% of all cases of schizophrenia^28^, and is more severely impaired in brain functions^29^. Compared with Zhou et al.’s^24^ study on early-onset schizophrenia sample, our findings in FES patients may be more generalizable to the clinical populations commonly encountered in early psychosis intervention services.

Previous evidence for atypical unisensory temporal processing in patients with ASD is mixed. For example, adults^30^ and toddlers^31^ with ASD were found to show superior visual temporal acuity compared to their typically developing counterparts, whereas other studies suggested children with ASD were less efficient in encoding temporal aspects of rapid auditory stimuli but not visual stimuli^32,33^. A recent meta-analysis has pooled relevant findings and reported that altered unisensory temporal processing is not a universal characteristic in patients with ASD, at least for visual stimuli^25^. Concurring with the previous work which utilized the same TOJ task as ours and failed to demonstrate any altered unisensory thresholds in ASD sample^24^, our negative findings in young adult sample further support the notion of intact unisensory temporal processing in ASD.

### Audiovisual temporal integration

This study shows widened TBW affecting both speech and non-speech processing in FES patients relative to controls, which coincides with previous evidence gathered from adult samples with chronic schizophrenia^18^ and also children and adolescents with early onset schizophrenia^24^. Altered audiovisual temporal integration may be a robust impairment associated with schizophrenia regardless of clinical stage and age range. Nonetheless, the between-group differences in the width of TBW are less pronounced in this study (medium effect size: Cohen’s *d* = 0.57–0.74) compared with earlier findings of large effect sizes (Cohen’s *d* > 0.8)^18,24^. This result indicates clinical characteristics such as cognitive abilities may at least partially explain schizophrenia patients’ worse performances in multisensory temporal processing.

A generalized inclination to integrate temporally discrete stimuli is the hallmark of altered temporal processing. In patients with schizophrenia, such altered temporal processing can manifest in different forms, such as their less precise time interval estimation and rhythm production^34^. In fact, it has been proposed that schizophrenia patients may have an altered “internal clock”^35^, yet more research is needed to clarify this postulation. On the other hand, given that multisensory temporal acuity shows a high degree of plasticity in the adult brains^8^ and perceptual trainings can potentially narrow the TBW width in healthy people^36^, future research may evaluate the effects of perceptual trainings on audiovisual temporal processing ability in schizophrenia patients.

As for adults with ASD, we found intact TBW for both speech and non-speech stimuli. Although the majority of evidence from children and adolescents with ASD suggests the presence of altered audiovisual temporal processing (see ref. ^8^ for a review), it is plausible that adults with ASD would have ameliorated TBWs^16,17^. This finding also concurs with meta-analytic findings which showed that the altered audiovisual integration would be more severe at young age^22^. In particular, ASD patients with normal intellectual function may eventually catch up with typically developing peers during adulthood, supporting the hypothesis of developmental delay in multisensory integration in ASD^37^. Future longitudinal research is needed to verify this postulation.

Our study did not find statistical difference between the TBWs of adults with FES and adults with ASD. This is a novel finding because no previous study had compared these patient groups directly. Decreased sensitivity in detecting audiovisual asynchrony has been proposed as a shared feature for ASD and schizophrenia^18^. When temporally-parsed sensory signals would be bound together excessively, this may result in improper perceptions in social encounters, and may undermine social communications in both disorders^24^. However, a previous study concerning adolescents with schizophrenia and children with ASD demonstrated more severe deficits of widened TBW in early onset schizophrenia compared to ASD^24^. One reason to explain the discrepancy may be related to our sample characteristics. Notably, our schizophrenia individuals were having first-episode and clinical stabilization, whereas Zhou et al.’s^24^ study utilized adolescent samples with more severe psychopathology.

### Correlations of temporal sensory processing with non-verbal IQ and symptoms

Our study found that lower non-verbal IQ was correlated with wider TBWs in adults with ASD. The correlational findings implicate the role of non-verbal IQ on temporal sensory processing. Sharpened multisensory temporal acuity has been found to predict better performances in non-verbal and verbal problem-solving tasks in healthy people^38^. Contrary to our findings, Meilleur et al.’s^25^ meta-analysis reported a non-significant correlation between IQ and temporal sensory processing in children with ASD. Although previous findings regarding the relationship between cognitive abilities and audiovisual temporal processing are mixed^25,38^, future research should measure several cognitive confounds, such as attention and memory, to examine whether specific differences of temporal processing exist beyond the generalized cognitive deficits associated with schizophrenia and ASD.

Our study did not find any correlation between TBW and symptom severity in patients with FES. However, several previous studies demonstrated an association between the severity of psychotic symptoms and TBW^13,15^. Reduced sensitivity to detect audiovisual speech asynchrony may also result in social misperceptions, when temporally discrete and unrelated social cues are integrated, thus contributing to more severe negative symptoms^24^. Our negative finding could be attributable to small sample size and low level of psychopathology in our sample with schizophrenia. More research is needed to clarify these issues, using larger adult samples with greater inter-individual variability in clinical symptoms.

### Limitations

Several limitations of this study should be born in mind. First, the diagnosis of ASD in our sample was not ascertained using the Autism Diagnostic Observation Schedule (ADOS). We also did not use symptoms rating scales to measure psychiatric symptoms in our ASD sample. Therefore, we could not investigate the correlation of unisensory and multisensory integration with ASD symptoms. The extant literature suggested inconsistent findings regarding the relationship between temporal processing and ASD symptom severity^25^, and future research is needed to clarify this area. Second, our schizophrenia group had lower non-verbal IQ than the other two groups. In fact, after entering non-verbal IQ as a covariate, our findings of enlarged TBW for non-speech and speech stimuli in schizophrenia patients became non-significant (see Supplementary Materials). Future research should employ refined paradigms to investigate temporal processing, and clarify the existence of any temporal processing impairment in schizophrenia which can be independent from generalized cognitive deficits. Although non-verbal IQ could influence the observed performances in our paradigms, lower IQ is considered an integral feature of schizophrenia, and controlling for schizophrenia patients’ lower non-verbal IQ may not enhance the generalizability of our findings^39^. Third, our sample size was relatively small, and the data of a considerable proportion of participants were excluded due to poor data-fitting or the minimum requirements of the paradigm. Having said that, the number of participants in all the tests exceeded the required sample size, even after some participants’ data were excluded. Fourth, all ASD participants had average IQ and relatively high-functioning, and therefore may undermine the generalizability of our findings to the ASD population. As for potential gender effects, although we recruited female participants with ASD (contrary to only male samples of ASD in all previous studies), the gender ratio in the ASD group was not balanced. On the other hand, such male predominance in ASD sample may represent the epidemiology of the ASD population (with a gender ratio of 3–4 male: 1 female)^40^. Fifth, this cross-sectional study could not reveal potential developmental changes of TBW in schizophrenia and ASD. The trajectory of altered audiovisual temporal integration in these two disorders should be further explored in future research. Moreover, different paradigms were used to examine temporal acuity in unisensory and multisensory modalities, but it is plausible that the TOJ and the SJ tasks may involve different aspects of temporal processing, with the TOJ task requiring “order” processing in addition to synchrony perception and thus eliciting stronger activity in several regions in the left hemisphere^41^. Our investigations of multisensory integration were only limited to the syllable level of speech, and this might have undermined the ecological validity of our SJ paradigm. Future studies may utilize more complex linguistic stimuli (e.g., short clips of everyday life conversations) to examine audiovisual speech processing in ASD and schizophrenia. Finally, apart from education, we did not cover comprehensive socioeconomic information such as socioeconomic status and family income.

## Conclusions

Our study is one of the few to investigate audiovisual temporal integration, and to directly compare the TBW between adult patients with schizophrenia and adult patients with ASD. Using sophisticated paradigms tapping into unisensory and audiovisual sensory integration, our findings suggested that FES patients exhibit widened audiovisual TBW relative to healthy people, but adults with ASD show largely preserved ability to perceptually integrate audiovisual signals based on temporal cues.

## Methods

### Participants

Our sample comprised adult patients with FES, verbally-fluent and average IQ (termed as “high-functioning ASD”) adult patients with ASD and controls. Participants with FES were recruited from the early psychosis intervention clinics at Castle Peak Hospital (CPH), Hong Kong; whereas participants with ASD were recruited from general adult psychiatric clinics at CPH. Controls were recruited from the neighboring community.

We estimated the sample size using the G*Power 3.1.9.7, and the previous meta-analytic findings regarding the deficits of audiovisual temporal processing in schizophrenia (Hedge’s g = 0.91) and ASD (Hedge’s g = 0.85)^18^. With an effect size of 0.8 (Cohen’s *d*), power of 0.8 and the significance level (alpha) of 0.05, the sample size needed to be >26 participants in each groups.

We recruited 43 participants with FES (aged 16–35) who fulfilled the diagnostic criteria of schizophrenia in accordance to the DSM-IV^42^ as ascertained by qualified psychiatrists using the Structured Clinical Interview for DSM-IV (SCID-I)^43^. All of them were receiving antipsychotic medications (27 monotherapy, 15 polypharmacy), and the mean olanzapine equivalence defined daily dose (DDD)^44^ was 14.24 mg/day (SD = 8.40 mg/day). Clinical symptoms were assessed using the Positive and Negative Syndrome Scale (PANSS)^45^.

In the ASD group, 35 participants (aged 18–35) were recruited, with the clinical diagnosis ascertained by qualified psychiatrists according to DSM-IV (as Pervasive Development Disorders) or DSM-5 (as ASD)^46^. Eleven of them were receiving antipsychotics (risperidone, *n* = 6; quetiapine, *n* = 1; aripiprazole, *n* = 4) (the mean olanzapine equivalence DDD = 3.63 mg/day, SD = 2.85 mg/day), and the remaining ASD participants were medication-free. As measured by the Extrapyramidal Side-effect Rating Scale (ESRS)^47^, all clinical participants (FES and ASD) receiving medications had low levels of extrapyramidal side effects (see Table 1).

We recruited 48 demographically-matched healthy individuals as controls. The SCID-I/NP was administered by qualified psychiatrists to ensure that controls were not having any diagnosable psychiatric disorder. For all the three groups of participants, exclusion criteria included (1) personal history of brain injury or neurological disorders; (2) a past history of substance use in the past 6 months; (3) mental retardation; (4) history of electroconvulsive therapy (ECT) in the past six months; and (5) other physical conditions that could interfere with performing the tests. For all the three groups of participants, inclusion criteria included (1) speaking native Cantonese (a southern dialect of Chinese); (2) normal hearing; and (3) normal or corrected-to-normal vision. All controls reported no biological first-degree relatives having DSM-IV Axis I mental disorders. Participants of the two clinical groups did not have any diagnosable co-morbid DSM-IV Axis I mental disorder. Participants with ASD did not have any syndromal or genetic disorders according to the medical records.

This study has been approved by the Clinical and Research Ethics Committee of New Territories West Cluster (NTWC) of Hong Kong (Protocol Number: NTWC/REC/20074). Written informed consent was obtained from all participants. The study was conducted from 1 July 2020 to 30 June 2021.

### Unisensory temporal order judgement (TOJ) tasks

To control for individual variations in unisensory temporal processing, we administered the computerized TOJ tasks^24^ to our sample. The TOJ tasks presented auditory or visual pairs in separate runs to participants. In the visual TOJ task, the stimuli were two white rings (outer diameter = 10 cm; thickness = 1.5 cm) presented on a black background on the computer screen, one above and one below the fixation cross (duration = 16.7 ms). In the auditory TOJ task, the stimuli consisted of a high- and low-pitch (2200 and 500 Hz, duration = 7 ms) pair of beep sound, presented via a headphone binaurally. In this study, unisensory SOAs ranged from 17 to 133 ms for visual stimuli (i.e., the SOA values = 16.7, 33.3, 50.0, 66.7, 83.4, 100.0, and 133.4 ms); and ranged from 17 to 250 ms for auditory stimuli (i.e., the SOA values = 16.7, 33.3, 50.0, 83.4, 100.0, 150.0, 200.0, and 250.0 ms). Participants were asked to judge which of the stimulus in the unisensory pair appeared first, by pressing specified buttons on a computer. Each SOA condition was repeated for 20 times and was presented randomly.

The SOA threshold at which a participant could attain 75% of accuracy in the TOJ tasks was estimated as a “proxy index” for his/her “unisensory temporal acuity”^26^. Participants’ whose TOJ accuracy lay 2 SD below the mean in the largest SOA condition were excluded. Details of the experimental design of the TOJ tasks have been described elsewhere^24^.

### Audiovisual simultaneity judgement (SJ) tasks

**Fig. 3:**
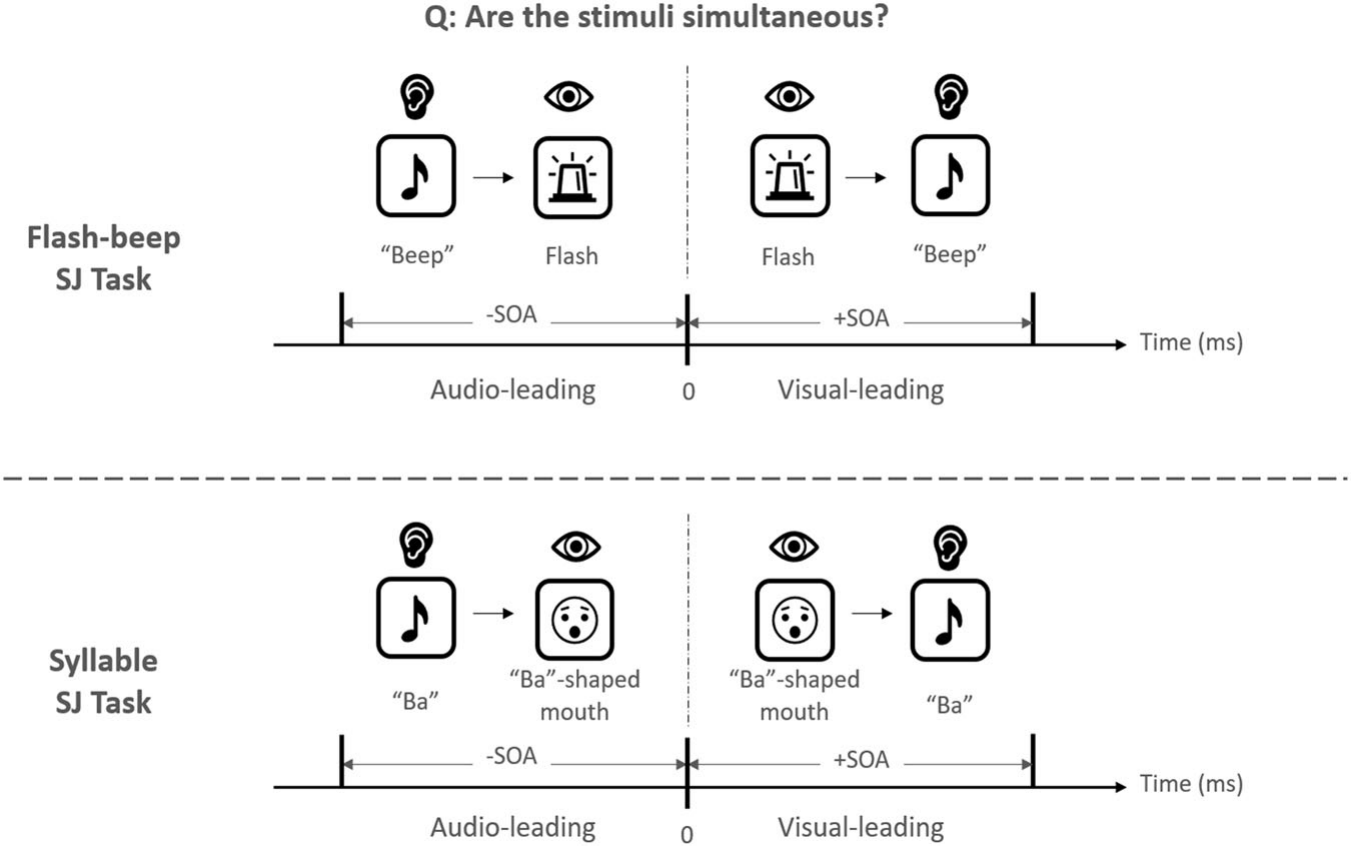
Illustration of flash-beep Simultaneity Judgement (SJ) task and syllable SJ task. Participants would be asked the question “Are the stimuli simultaneous?” in the SJ tasks. SOA Stimulus Onset Asynchronies (in milliseconds), Audio-leading (AL) pairs have negative values of SOA and visual-leading (VL) pairs have positive values of SOA.

The computerized SJ tasks comprised two sessions, i.e., the flash-beep SJ task and the syllable SJ task, which measured participants’ TBW for non-speech and speech stimuli respectively^24^. In the flash-beep SJ task, a flash stimulus (i.e., a white circle with a radius of 15 cm and thickness of 4 cm at the center of the computer screen, duration = 16.7 ms) and a beep sound (at 1000 Hz, duration = 16.7 ms) would be presented sequentially, with thirteen pre-defined SOA conditions ranged from +600 ms to −600 ms in 100 ms intervals (see Fig. 3). In the syllable task, a visual stimulus of a short video clip filming a native Cantonese female speaker who opened her mouth as if she was speaking, and a sound of the single syllable “ba” would be played sequentially. The speaker maintained a flat prosody and neutral facial expression throughout the video. The thirteen pre-defined SOA conditions ranged from +720 ms to −720 ms in 120 ms intervals (see Fig. 3).

The two SJ tasks comprised equal numbers of trials of audio-leading (AL) pairs (i.e., having negative values of SOA) and visual-leading (VL) pairs (i.e., having positive values of SOA). Participants were asked to report whether the auditory and visual stimuli were perceived as synchronous or not, by pressing the specified buttons on the keyboard. Each SOA condition was repeated for 10 times, and the trials were randomly presented to participants. A total of 130 trials in total for the flash-beep and syllable SJ tasks was presented.

The data regarding the proportion of trials of synchrony responses for each SOA condition were calculated, and then applied to fitting a Gaussian distribution model on a participant-by-participant basis. Following our previous method^24^, we estimated the standard deviation (SD) of the Gaussian function, which was regarded as an estimate of the width of TBW. Moreover, we estimated the mean of the Gaussian function to identify the specific SOA at which a participant exhibited the highest likelihood to perceive stimuli as synchronous (i.e., the “point of subjective simultaneity”, PSS). If a participant’s data gathered in SJ tasks fit very poorly (*R*^2^ < 0.3) to the Gaussian distribution model, he/she would be excluded from further data analysis. Details of the experimental design of the SJ tasks have been described elsewhere^24^.

### Non-verbal IQ estimation

The Test of Nonverbal Intelligence - Fourth Edition (TONI-4)^48^ was administered to all participants by trained research assistants. The TONI-4 consists of 60 items in abstract or figural formats. The TONI-4 was used because this IQ measure would unlikely be affected by participants’ educational, cultural and experiential backgrounds, and would be suitable for ASD samples. In this study, the inter-rater reliability among the three assessors had reached 0.99.

### Procedure

Participants were first interviewed by qualified psychiatrists to ascertain clinical diagnosis and assess symptom severity and non-verbal IQ. Then, we administered the TOJ and SJ paradigms in a dimly lit, sound-attenuated room. Visual stimuli were presented using a 19-inch Cathode Ray Tube (CRT) screen (60 Hz), and auditory stimuli were presented using a headphone placed binaurally to the participants. Before each formal task, we adjusted the sound volume on a participant-by-participant basis, to ensure that he/she could hear the auditory signals clearly. We controlled the stimulus presentation using the Psychtoolbox extension in Matlab. Before each formal task, participants had completed 10 practice trials (which had the least difficult SOA conditions) to make sure that they could understand the task instructions correctly. Participants must have achieved an accuracy of 75% or above in the practice sessions, before undergoing the formal tasks. Participants who failed to achieve the minimum accuracy of 75% in practice trials were excluded from further analyses. We chose the range of SOA conditions in the TOJ and SJ tasks based on our pilot data, to ensure that the majority of participants could achieve a high accuracy in the practice trials, could follow the task instructions, and could successfully detect asynchronies at the largest SOAs. The SJ and TOJ task each took 5–10 min to complete. To minimize participants’ likelihood of getting fatigue, we divided the paradigm into four blocks, for both the SJ tasks (flash-beep, syllable) and the TOJ tasks (visual, auditory). Participants were allowed to have breaks as long as needed during the intervals between the blocks of the four tasks. The participant could also take breaks between the tasks.

### Data analysis

To examine group differences in performances of the TOJ tasks, we entered the accuracy scores into a mixed model ANOVA, with Group (schizophrenia, ASD, controls) as the between-group variable, and the SOA condition as the within-group variable. Likewise, for the SJ tasks, we entered the percentage of perceived synchrony into a mixed model ANOVA, with Group as the between-group variable, and the SOA condition as the within-group variable. The Group main effect and the Group-by-SOA condition interaction effect were estimated to indicate participant groups’ patterns of unisensory and multisensory temporal acuity.

We examined group differences in (1) unisensory TOJ threshold, and (2) audiovisual TBW width, and (3) PSS (for speech and non-speech stimuli) using ANOVA, with post-hoc (Sidak) comparison. To examine the relationship between temporal processing and clinical characteristics, the TOJ threshold and TBW were correlated with estimated non-verbal IQ, medication dosage (in DDD), extra-pyramidal side-effects, and psychopathological symptoms (the PANSS subscale scores) using Spearman’s correlations (Spearman’s rho). Spearman’s correlations were first calculated across the three groups, and then within each group of participants. Because of our relatively small sample size, adjustments for multiple hypothesis testing were not applied to the correlational analysis.

## Supplementary information

Supplementary Materials
