# Supplementary material for: Audiovisual temporal processing in adult patients with first-episode schizophrenia and high-functioning autism
Source: Schizophrenia (Heidelb). 2022 Sep 22;8(1):75. doi: 10.1038/s41537-022-00284-2 (PMC9500036; doi:10.1038/s41537-022-00284-2)
Supplement: Supplementary file 1 — Supplementary Materials [file 41537_2022_284_MOESM1_ESM.docx]

**Supplementary Materials**

**Supplementary Data Analysis and Findings**

**(1) Comparison of age and gender ratio across the three groups after excluding participants who failed to pass practice trials or showed poor data-fitting**

As a considerable proportion of participants were excluded due to poor data-fitting or the minimum requirements of the paradigm, we further examined whether age and gender would still be equivalent between groups after exclusion of participants.

For the visual TOJ task, 41 participants with FES, 29 ASD participants and 45 controls were included to compare the visual temporal thresholds. The three groups were matched in age (*F*_[2, 112]_ = 2.48 , *p* =.09) and gender ratio (*χ*_[2]_ = 1.98, *p* = .37).

For the auditory TOJ task (36 participants with FES, 29 ASD participants and 42 controls), the three groups of participants included for auditory temporal acuity comparison showed comparable gender ratio (*χ*_[2]_ = 1.73, *p* = .42). However, they differed significantly in age (*F*_[2, 104]_ = 3.23, *p* = .044), with FES participants (mean age = 26.56, S.D. = 5.25) older than ASD participants (mean age = 23.79, S.D. = 4.06) (*p* < .05).

For the audiovisual SJ task for non-speech stimuli, the remaining participants included for estimating TBW (34 participants with FES, 30 ASD participants and 48 controls) showed comparable age (*F*_[2, 109]_ = 1.50, *p* = .23) and gender ratio (*χ*_[2]_ = 0.99, *p* = .61) across the three groups. Similarly, in the audiovisual SJ task for speech stimuli, the three groups of participants included (31 participants with FES, 29 ASD participants and 46 controls) were well-matched in age (*F*_[2, 103]_ = 1.77, *p* = .18) and gender (*χ*_[2]_ = 0.72, *p* = .70).

**(2) Analysis of group differences in audiovisual TBW with IQ as a covariate**

In view of the low non-verbal IQ score in the FES group, we further used ANCOVA to examine the group differences in audiovisual temporal integration with IQ as a covariate. Non-verbal IQ was found to be a significant covariate in the model for flash-beep TBW (*F*_[1,108]_ = 11.378, *p* = 0.001, partial-η^2^ = 0.095) and syllable TBW (*F*_[1,102]_ = 12.092, *p* = 0.001, partial-η^2^ = 0.126). The previous significant group differences disappeared in the flash-beep TBW (*F*_[2,108]_ = 1.023, p = 0.363, partial-η^2^ = 0.019) and syllable TBW (*F*_[1,102]_ = 0.928, p = 0.399, partial-η^2^ = 0.018), after controlling for group difference in IQ.

**Supplementary Table 1. The group differences in TOJ accuracy scores in each of the SOA conditions in the visual TOJ task**

|  | FEP | | ASD | | Controls | |  |  | Post-hoc (Sidak) | | | | | |
| --- | --- | --- | --- | --- | --- | --- | --- | --- | --- | --- | --- | --- | --- | --- |
| SOA | **n = 43** | | **n = 35** | | **n = 48** | |  |  | **FEP vs controls** | | **ASD  vs control** | | **FEP vs ASD** | |
| Condition | **mean** | **SD** | **mean** | **SD** | **mean** | **SD** | ***F*_[2,123]_** | ***p*** | ***p*** | **Cohen's *d*** | ***p*** | **Cohen's *d*** | ***p*** | **Cohen's *d*** |
| 133 | 0.964 | 0.080 | 0.891 | 0.220 | 0.988 | 0.032 | 6.128 | 0.003* | - | - | 0.001 | 0.62 | 0.013 | 0.44 |
| 100 | 0.957 | 0.080 | 0.883 | 0.220 | 0.977 | 0.055 | 5.726 | 0.004* | - | - | 0.001 | 0.59 | 0.013 | 0.45 |
| 83 | 0.948 | 0.088 | 0.893 | 0.214 | 0.963 | 0.081 | 2.934 | 0.057 | - | - | 0.020 | 0.43 | - | - |
| 67 | 0.936 | 0.093 | 0.879 | 0.214 | 0.960 | 0.071 | 3.938 | 0.022* | - | - | 0.006 | 0.51 | - | - |
| 50 | 0.855 | 0.130 | 0.836 | 0.222 | 0.881 | 0.136 | 0.820 | 0.443 | - | - | - | - | - | - |
| 33 | 0.871 | 0.133 | 0.851 | 0.198 | 0.898 | 0.111 | 1.052 | 0.352 | - | - | - | - | - | - |
| 17 | 0.695 | 0.144 | 0.709 | 0.172 | 0.721 | 0.157 | 0.298 | 0.743 | - | - | - | - | - | - |

**Note.** SOA = Stimulus Onset Asynchrony (in ms); FEP = first-episode schizophrenia; ASD = Autism Spectrum Disorder; “*” indicates *p* < 0.05; Post-hoc Sidak adjustments were only performed in SOA conditions with significant difference.

**Supplementary Table 2. The group differences in TOJ accuracy scores in each of the SOA conditions in the auditory TOJ task**

|  | FEP | | ASD | | Controls | |  |  | Post-hoc (Sidak) | | | | | |
| --- | --- | --- | --- | --- | --- | --- | --- | --- | --- | --- | --- | --- | --- | --- |
| SOA | **n = 42** | | **n = 35** | | **n = 48** | |  |  | **FEP vs controls** | | **ASD  vs controls** | | **FEP vs ASD** | |
| Condition | **mean** | **SD** | **mean** | **SD** | **mean** | **SD** | ***F*_[2,122]_** | ***p*** | ***p*** | **Cohen's *d*** | ***p*** | **Cohen's *d*** | ***p*** | **Cohen's *d*** |
| 250 | 0.838 | 0.220 | 0.843 | 0.249 | 0.882 | 0.250 | 0.456 | 0.635 | - | - | - | - | - | - |
| 200 | 0.833 | 0.231 | 0.849 | 0.236 | 0.885 | 0.235 | 0.591 | 0.555 | - | - | - | - | - | - |
| 150 | 0.830 | 0.238 | 0.836 | 0.247 | 0.877 | 0.259 | 0.481 | 0.619 | - | - | - | - | - | - |
| 100 | 0.818 | 0.245 | 0.836 | 0.238 | 0.882 | 0.246 | 0.841 | 0.434 | - | - | - | - | - | - |
| 83 | 0.808 | 0.204 | 0.840 | 0.219 | 0.876 | 0.232 | 1.074 | 0.345 | - | - | - | - | - | - |
| 50 | 0.754 | 0.235 | 0.816 | 0.230 | 0.854 | 0.239 | 2.066 | 0.131 | - | - | - | - | - | - |
| 33 | 0.710 | 0.222 | 0.751 | 0.219 | 0.788 | 0.215 | 1.425 | 0.245 | - | - | - | - | - | - |
| 17 | 0.650 | 0.197 | 0.683 | 0.191 | 0.673 | 0.193 | 0.320 | 0.727 | - | - | - | - | - | - |

**Note.** SOA = Stimulus Onset Asynchrony (in ms); FEP = first-episode schizophrenia; ASD = Autism Spectrum Disorder; “*” indicates *p* < 0.05; Post-hoc Sidak adjustments were only performed in SOA conditions with significant difference.

**Supplementary Table 3. The group differences in perceived synchrony scores in each of the SOA conditions in the flash-beep SJ task**

|  | FEP | | ASD | | Controls | |  |  | Post-hoc (Sidak) | | | | | |
| --- | --- | --- | --- | --- | --- | --- | --- | --- | --- | --- | --- | --- | --- | --- |
| SOA | **n = 35** | | **n = 35** | | **n = 48** | |  |  | **FEP vs controls** | | **ASD  vs controls** | | **FEP vs ASD** | |
| Condition | **mean** | **SD** | **mean** | **SD** | **mean** | **SD** | ***F*_[2,115]_** | ***p*** | ***p*** | **Cohen's *d*** | ***p*** | **Cohen's *d*** | ***p*** | **Cohen's *d*** |
| -600 | 0.206 | 0.230 | 0.166 | 0.234 | 0.073 | 0.145 | 4.824 | 0.010* | 0.011 | 0.69 | - | - | - | - |
| -500 | 0.300 | 0.247 | 0.189 | 0.236 | 0.150 | 0.168 | 5.108 | 0.007* | 0.006 | 0.710 | - | - | - | - |
| -400 | 0.397 | 0.233 | 0.243 | 0.257 | 0.267 | 0.275 | 3.744 | 0.027* | - | - | - | - | 0.041 | 0.629 |
| -300 | 0.617 | 0.270 | 0.374 | 0.277 | 0.427 | 0.264 | 8.030 | 0.001* | 0.006 | 0.712 | - | - | 0.001 | 0.889 |
| -200 | 0.814 | 0.196 | 0.671 | 0.218 | 0.719 | 0.209 | 4.326 | 0.015* | - | - | - | - | 0.014 | 0.690 |
| -100 | 0.940 | 0.098 | 0.871 | 0.167 | 0.915 | 0.109 | 2.648 | 0.075 | - | - | - | - | - | - |
| 0 | 0.949 | 0.082 | 0.891 | 0.165 | 0.942 | 0.087 | 2.682 | 0.073 | - | - | - | - | - | - |
| +100 | 0.914 | 0.100 | 0.831 | 0.201 | 0.929 | 0.122 | 5.005 | 0.008* | - | - | 0.009 | -0.588 | - | - |
| +200 | 0.834 | 0.153 | 0.669 | 0.251 | 0.798 | 0.220 | 5.990 | 0.003* | - | - | 0.022 | -0.549 | 0.004 | 0.798 |
| +300 | 0.706 | 0.273 | 0.543 | 0.296 | 0.552 | 0.284 | 3.786 | 0.026* | 0.050 | 0.552 | - | - | - | - |
| +400 | 0.557 | 0.288 | 0.409 | 0.326 | 0.398 | 0.291 | 3.264 | 0.042* | - | - | - | - | - | - |
| +500 | 0.417 | 0.297 | 0.349 | 0.336 | 0.204 | 0.223 | 6.213 | 0.003* | 0.003 | 0.812 | - | - | - | - |
| +600 | 0.329 | 0.270 | 0.297 | 0.348 | 0.183 | 0.236 | 3.091 | 0.049* | - |  | - | - | - | - |

**Note.** SOA = Stimulus Onset Asynchrony (in ms); FEP = first-episode schizophrenia; ASD = Autism Spectrum Disorder; “*” indicates *p* < 0.05; Post-hoc Sidak adjustments were only performed in SOA conditions with significant difference.

**Supplementary Table 4. The group differences in perceived synchrony scores in each of the SOA conditions in the syllable SJ task**

|  | FEP | | | ASD | | | Controls | |  | | | | Post hoc (Sidak) | | | | | | | | | | |
| --- | --- | --- | --- | --- | --- | --- | --- | --- | --- | --- | --- | --- | --- | --- | --- | --- | --- | --- | --- | --- | --- | --- | --- |
| SOA | **n = 32** | | | **n = 34** | | | **n = 46** | | | |  |  | | **FEP vs controls** | | | | **ASD vs controls** | | | **FEP vs ASD** | |  |
| Condition | **mean** | **SD** | **mean** | | **SD** | **mean** | | **SD** | | ***F*_[2,109]_** | | ***p*** | | | ***p*** | **Cohen's *d*** | ***p*** | | **Cohen's *d*** | ***p*** | | **Cohen's *d*** |  |
| -720 | 0.378 | 0.359 | 0.285 | | 0.296 | 0.152 | | 0.232 | | 5.878 | | 0.004* | | | 0.003 | 0.478 | - | | - | - | | - |  |
| -600 | 0.488 | 0.389 | 0.350 | | 0.360 | 0.241 | | 0.282 | | 4.981 | | 0.009* | | | 0.006 | 0.425 | - | | - | - | | - |  |
| -480 | 0.547 | 0.403 | 0.388 | | 0.357 | 0.317 | | 0.290 | | 4.207 | | 0.017* | | | 0.014 | 0.653 | - | | - | - | | - |  |
| -360 | 0.747 | 0.352 | 0.597 | | 0.362 | 0.728 | | 0.298 | | 2.075 | | 0.131 | | | - | - | - | | - | - | | - |  |
| -240 | 0.844 | 0.278 | 0.762 | | 0.329 | 0.883 | | 0.182 | | 2.110 | | 0.126 | | | - | - | - | | - | - | | - |  |
| -120 | 0.956 | 0.122 | 0.853 | | 0.257 | 0.954 | | 0.084 | | 4.557 | | 0.013* | | | - | - | 0.023 | | -0.530 | 0.037 | | 0.513 |  |
| 0 | 0.981 | 0.047 | 0.891 | | 0.252 | 0.989 | | 0.031 | | 5.290 | | 0.006* | | | - | - | 0.009 | | -0.547 | 0.034 | | 0.498 |  |
| +120 | 0.956 | 0.095 | 0.862 | | 0.234 | 0.941 | | 0.124 | | 3.502 | | 0.034* | | | - | - | - | | - | - | | - |  |
| +240 | 0.847 | 0.224 | 0.644 | | 0.331 | 0.657 | | 0.301 | | 5.168 | | 0.007* | | | 0.016 | 0.717 | - | | - | 0.017 | | 0.717 |  |
| +360 | 0.438 | 0.330 | 0.250 | | 0.287 | 0.302 | | 0.284 | | 3.474 | | 0.034* | | | - | - | - | | - | 0.037 | | 0.606 |  |
| +480 | 0.328 | 0.343 | 0.209 | | 0.276 | 0.137 | | 0.208 | | 4.648 | | 0.012* | | | 0.009 | 0.674 | - | | - | - | | - |  |
| +600 | 0.141 | 0.218 | 0.168 | | 0.294 | 0.057 | | 0.128 | | 2.932 | | 0.057 | | | - | - | - | | - | - | | - |  |
| +720 | 0.109 | 0.215 | 0.147 | | 0.258 | 0.054 | | 0.133 | | 2.138 | | 0.123 | | | - | - | - | | - | - | | - |  |

**Note.** SOA = Stimulus Onset Asynchrony (in ms); FEP = first-episode schizophrenia; ASD = Autism Spectrum Disorder; “*” indicates *p* < 0.05; Post-hoc Sidak adjustments were only performed in SOA conditions with significant difference.

**Supplementary Table 5**. **Spearman correlations between temporal acuity, non-verbal IQ and clinical features within and across groups**

| Group |  | Visual TOJ threshold | Auditory TOJ threshold | Flash-beep TBW | Syllable TBW |
| --- | --- | --- | --- | --- | --- |
| FES | Non-verbal IQ | -0.008 (*p* = .96) | -0.311 (*p* = .07) | -0.102 (*p* = .57) | -0.233 (*p* = .21) |
|  | PANSS positive | 0.077 (*p* = .63) | 0.101 (*p* = .56) | -0.059 (*p* = .74) | 0.152 (*p* = .41) |
|  | PANSS negative | -0.178 (*p* = .27) | 0.166 (*p* = .33) | 0.169 (*p* = .34) | 0.197 (*p* = .29) |
|  | PANSS general | -0.040 (*p* = .80) | 0.132 (*p* = .44) | 0.083 (*p* = .64) | 0.174 (*p* = .35) |
|  | DDD | 0.020 (*p* = .90) | 0.111 (*p* = .52) | 0.167 (*p* = .35) | -0.028 (*p* = .88) |
|  | ESRS | 0.018 (*p* = .91) | 0.087 (*p* = .62) | 0.062 (*p* = .73) | 0.143 (*p* = .44) |
| ASD | Non-verbal IQ | -0.035 (*p* = .86) | -0.076 (*p* = .70) | **-0.416^*^(*p* = .02)** | **-0.380^*^(*p* = .04)** |
| Controls | Non-verbal IQ | -0.186 (*p* = .22) | 0.043 (*p* =.79) | -0.199 (*p* = .18) | -0.118 (*p* = .44) |
| Entire sample | Non-verbal IQ | -0.072 (*p* = .45) | -0.124 (*p* = .20) | **-0.409^**^(*p* < .001)** | **-0.329^**^(*p* = .001)** |

**Note.** ^*^*p* < .05; ^**^*p* < .01; FES = first-episode schizophrenia; ASD = autism spectrum disorder; PANSS = The Positive and Negative Syndrome Scale; DDD = Defined Daily Dose (mg/day; olanzapine equivalence); ESRS = Extrapyramidal Symptom Rating Scale.
